# Numerical Study on Mechanical Responses during Quench Protection in High-Temperature Superconducting Coils

**DOI:** 10.3390/ma16124356

**Published:** 2023-06-13

**Authors:** Ruoshan Jiao, Mingzhi Guan

**Affiliations:** 1 Advanced Energy Science and Technology Guangdong Laboratory, Huizhou 516000, China; jiaorsh20@lzu.edu.cn; 2 Key Laboratory of Mechanics on Western Disaster and Environment, Ministry of Education, College of Civil Engineering and Mechanics, Lanzhou University, Lanzhou 730000, China; 3 Institute of Modern Physics, Chinese Academy of Sciences, Lanzhou 730000, China

**Keywords:** REBCO insulation coil, quench protection, mechanical responses, radial strain rate

## Abstract

In this paper, mechanical responses and electro-thermal characteristics of a rare earth barium copper oxide (REBCO) high-temperature superconducting (HTS) insulated pancake coil during the quenching process are investigated through finite element modeling (FEM). Firstly, a two-dimensional axisymmetric electro–magneto–thermal–mechanical FEM model with real dimensions is developed. Based on the FEM model, a systematic study on the effects of the time taken to trigger the system dump, background magnetic field, material properties of constituent layers, and coil size on quench behaviors of an HTS-insulated pancake coil is implemented. The variations in the temperature, current, and stress–strain in the REBCO pancake coil are studied. The results indicate that an increase in the time taken to trigger the system dump can increase the peak temperature of the hot spot but has no influence on the dissipation velocity. An apparent slope change of the radial strain rate is observed when the quench occurs regardless of the background field. During quench protection, the radial stress and strain reach their maximum values and then decrease as the temperature decreases. The axial background magnetic field has a significant influence on the radial stress. Measures to reduce peak stress and strain are also discussed, which indicates that increasing the thermal conductivity of the insulation layer, copper thickness, and inner coil radius can effectively reduce the radial stress and strain.

## 1. Introduction

Since their discovery in 1986 [1], high-temperature superconducting (HTS) materials have shown significant potential in many cutting-edge scientific fields and high-power electromagnetic applications such as particle accelerators in high-energy physics, magnetic resonance imaging (MRI), power cables, and superconducting motors [2,3,4,5]. Rare earth barium copper oxide (REBCO)-coated conductors are one of the best practical conductors for HTS coils due to their high current density under high magnetic fields and mechanical strength [6]. When HTS coils operate in the superconducting state, they can conduct a higher amount of current, but the loss is much smaller than that of normal metal conductors. In addition, for superconducting motors and magnets, HTS coils can greatly increase magnetic flux density, which leads to improvement in overall efficiency and reductions in weight and size. It is precisely due to these advantages that the stunning record of a DC magnet field of 45.5 T has been obtained by assembling a REBCO-coated conductor winding generating a magnetic field of 14.4 tesla inside a 31.1 tesla resistive background magnet [7]. Generally, REBCO coils operate under extremely low temperatures and high background magnetic fields. A quench phenomenon may occur in REBCO coils because of thermal disturbances, overcurrent processes, nonrecoverable local defects, and other factors [8]. Localized high-temperature gradients and temperature rises may be caused by the low normal zone propagation velocity (NZPV) of REBCO insulation coils. The rise in temperature in these coils, the internal voltages developed, and the large thermal stress and high Lorentz force during the quench process are critical issues for coil safety and can cause permanent magnet damage; for example, the European Organization for Nuclear Research Large Hadron Collider suffered electrical and mechanical damage due to quench [9].

In superconducting magnets, quench characterization is relatively complicated. The simulation of quench can help us to understand the quench propagation and behavior inside a magnet and to design effective quench detection and protection methods. To date, a number of previous research works have reported an HTS quench model that accounts for multi-field coupling. Based on a micrometer-scale mixed-dimensional REBCO tape model, Chan et al. used a hierarchical three-dimensional (3D) multiscale electro–magneto–thermal model of quench to study the electrical, thermal, and structural behavior in REBCO coils. Using their model, the local and global temperature and voltage profiles of a coil can be obtained [10,11]. Breschi et al. considered the distributed thermal resistance in the axial direction and built a two-dimensional (2D) electro–thermal nonlinear finite element modeling (FEM) model within the framework of a quasi-3D FEM model [12]. Their simulation results agreed well with measured values. Considering the uncertainty in evaluating the critical current and the local inhomogeneities of the critical current along an HTS tape, Badel et al. proposed an electro–thermal model to simulate the development and propagation of dissipative zones in a REBCO coil [13]. Zeng et al. investigated the temperature field distributions of a REBCO pancake coil using an electromagnetic–superconducting coupled FEM method [14]. This model can describe the nonlinear relation between the highest temperature and the current inside a coil.

With the present intense research focus on HTS magnets, electro–magneto–thermal modeling work has shown considerable progress. Johansen indicated that the mechanical characteristics of HTS materials’ response to high magnetic fields might be more important than their critical current density [15]. Studies show that REBCO conductors are sensitive to various kinds of mechanical deformation, and excessive stress/strain will cause structural failure and electro-mechanical degradation [16,17,18,19,20,21,22,23,24]. In practical applications, REBCO superconducting coils are subjected to thermal residual stress and strong hoop electromagnetic forces when operating under cryogenics and high-field [25,26,27,28,29,30].

During quench propagation, enormous thermal stresses/strains are generated due to the thermal mismatch and huge temperature gradient. Under the combined action of a huge electromagnetic force and thermal stress, the electrical properties and structure of HTS magnets may be affected. The critical current of the magnet will drop severely when the stress exceeds the corresponding yield stress [31]. Furthermore, a REBCO tape has a high axial tensile strength, but its interfacial strength is as low as 2–100 MPa [32,33,34,35], and its cleavage strength and peel strength are less than 1 MPa [36]. Therefore, delamination between the constituent layers may occur if the stress exceeds the corresponding critical value. The study of mechanical responses has been recognized to be important, and many scholars have examined the mechanical responses of HTS magnets. For instance, based on the generalized thermoelastic theory, Tong et al. studied the thermal–mechanical characteristics of superconducting composite tapes [37]. In their model, we find that the strain rate is strongly linked to the quench. However, plastic deformation in the REBCO tape may occur due to a high electromagnetic force and thermal stress. Niu et al. established an electro–magneto–thermal and elastoplastic mechanical model and applied this model to analyze the stress and strain distributions in a coil [38,39]. Quench protection for superconducting magnets can be implemented either with active or passive methods. The passive quench protection method is used for HTS coils with low stored energies, while active quench protection is suitable for large HTS coils [40,41,42]. No-insulation (NI) HTS coil is one of the popular solutions for quench protection, which is self-protection [43,44]. The dynamic current distribution, transient voltage, temperature profiles, and mechanical behavior of HTS coils have been addressed by different research groups [45,46,47,48,49,50,51]. Although much research on the mechanical behavior of HTS magnets in the process of winding, excitation, cooling, and quenching has been conducted, the reliability and safety evaluation of the superconducting structures during quench protection is still rarely involved. During the process of quench protection, the mechanical responses of HTS coils and the relationships with the electricity and heat need to be further explored. The huge unbalanced Lorentz force and thermal stress during the quench process may cause structural deformation, on the one hand, and performance degradation of superconducting materials, on the other hand, such as critical current degradation. Therefore, it is of great significance for the quench detection and protection of the superconducting magnets to study the thermal stability, electrical behaviors, and mechanical responses.

The objective of this paper is to study the mechanical responses and electro–thermal characteristics of a REBCO pancake coil during quench protection. In this paper, based on the extreme operating conditions of superconducting magnets, the quench protection of the HTS coil is studied from the perspective of multiple physical fields. The quench and characteristics and internal correlation of temperature, current, and mechanical response in the coils during quench propagation and quench protection are analyzed, which is the basis of the quench detection and protection research of superconducting magnets. The reliability and safety evaluation of the structure of superconducting magnets during quench protection is preliminarily explored. This paper is organized as follows. In Section 2, the details of the electro–magneto–thermal model and elastic mechanical model are described. In Section 3.1, the influence of the time taken to trigger the system dump and the material properties of the insulation layer on the mechanical responses and electro–thermal characteristics during quench protection are discussed. The mechanical responses of a pancake coil under a strong background magnetic field are investigated, as well. In Section 3.2, potential measures to reduce peak stress and strain are presented. Finally, the main conclusions are given in Section 4.

## 2. Model Description

**Figure 1:**
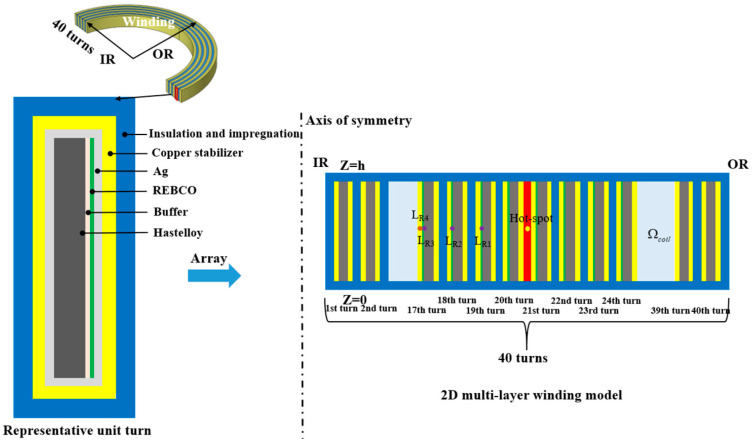
Schematic diagram of the REBCO pancake coil, where HTS tapes are separated by Kapton tape. Three probes, *L_R_*_1_, *L_R_*_2_, and *L_R_*_3_, are arranged on the REBCO layers, and one probe, *L_R_*_4_, is placed on the copper stabilizer layer to observe the changes in temperature and stress–strain. The quench is triggered by the heat power, which is uniformly exerted in the Kapton layer of the 20th turn, “*Ω_coil_*” denotes the coil domain, and “*IR*” and “*OR*” represent the positions of the inner radius and outer radius of the pancake coil, respectively.

The object in our simulation is an insulated pancake coil with a turn number of 40, fabricated by co-winding REBCO HTS tape and Kapton insulation. The HTS tape is mainly composed of a REBCO superconducting film, Hastelloy substrate, buffer layer, silver layers, and copper stabilizer. The composition of the REBCO layer, silver layer, and buffer layer is very small. The thermal and electrical properties of the tape are mainly controlled by the copper layers and Hastelloy layer. In the modeling process, to speed up the calculation, most of the constituent layers are considered, while the buffer layers, silver layers, and partial REBCO layers are omitted. Considering the axisymmetric property of the geometric structure and the physical field distribution of the coil, a reduced 2D axisymmetric model can be established with realistic dimensions, as shown in Figure 1, where the height, inner radius, and turn number of the coil are 4 mm, 30 mm, and 40, respectively. The thickness of the REBCO, copper stabilizer, Hastelloy substrate, and Kapton are 1 μm, 20 μm, 50 μm, and 59 μm, respectively. In the simulation, we assume that the quench is triggered by the heat power, which is uniformly exerted in the Kapton layer of the 20th turn for 0.02 s, with an input energy of 10 W.

In this work, multi-field coupled analysis is implemented in the finite element software COMSOL Multiphysics. The electromagnetic, thermal, mechanical, and electrical circuit models are used to calculate the hot spot temperature, current, and mechanical responses. In the quench process, the current density of REBCO will undergo drastic changes with temperature. The resistivity of the superconducting layer can be calculated from the E-J power and is considered a control parameter in the thermal–mechanical equations.

### 2.1. Magneto–Thermal–Mechanical

Maxwell’s equations can be used to describe electromagnetic phenomena at a macroscopic scale. In this section, we calculate the electromagnetic field distribution of the coil with H-formulation [52].

(1) $$\nabla \times E+\frac{\partial B}{\partial t}=0$$

**Figure 2:**
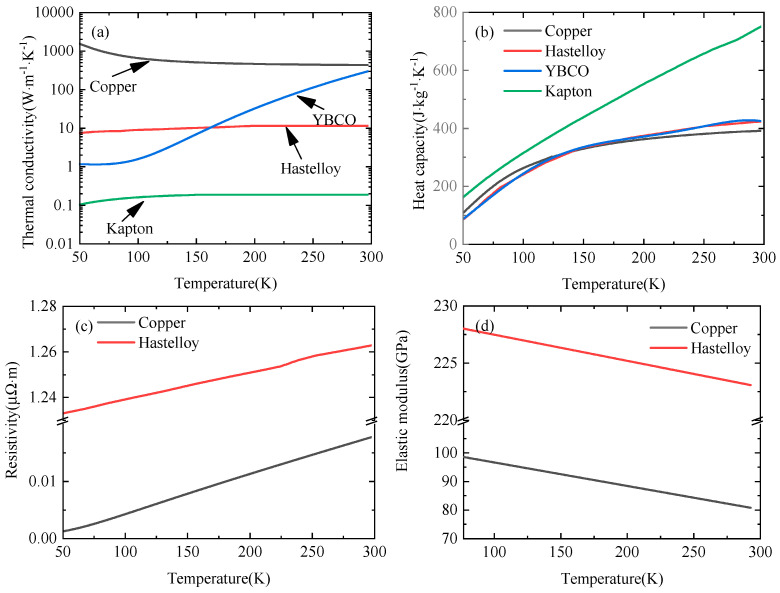
The (**a**) thermal conductivity, (**b**) specific heat capacity, (**c**) resistivity, and (**d**) elastic modulus, which are temperature-dependent [53,54,55,56,57,58,59].

(2) ∇×H=J

where **E** is the electric field; **B** is the magnetic flux density; *t* is the time; **H** is the magnetic field intensity; **J** is the current density. Combining electromagnetic constitutive relationship **E** = *ρ***J** and **B** = *μ*_0_*μ_r_***H**, the 2D axisymmetric governing equations are written as follows:

(3) $$\left\{\begin{array}{c} \mu_{0}\mu_{r}r\frac{\partial H_{r}}{\partial t}-\frac{\partial }{\partial z}\left[r\rho \left(\frac{\partial H_{r}}{\partial z}-\frac{\partial H_{z}}{\partial r}\right)\right]=0\\ \mu_{0}\mu_{r}r\frac{\partial H_{z}}{\partial t}+\frac{\partial }{\partial r}\left[r\rho \left(\frac{\partial H_{r}}{\partial z}-\frac{\partial H_{z}}{\partial r}\right)\right]=0 \end{array}\right.$$

where *μ*_0_ is the magnetic permeability, and *μ_r_* is the relative permeability. The value of *μ_r_* is set as 1. *r* and *z* are the radial and axial directions, respectively. *H_r_* and *H_z_* are the radial and axial magnetic field intensities, respectively. *ρ* is the material resistivity, which is temperature-dependent. The resistivity value of air is set as 1 Ω·m. The resistivity values of copper and Hastelloy are provided in Figure 2 [53,54,55,56,57,58,59].The resistivity of the REBCO layers is as follows [60]:

(4) $$\rho =\left\{\begin{array}{c} \frac{\rho_{S}\rho_{norm}}{\rho_{S}+\rho_{norm}},T<T_{c}\\ \rho_{norm},T\geq T_{c} \end{array}\right.$$

where *T_c_* is the critical temperature, and *T* is the local temperature. *ρ_norm_* =3 × 10^−6^ Ω·m [60] represents the REBCO resistivity when *T* ≥ *T_c_*, *ρ*_s_ represents the REBCO resistivity when *T* < *T_c_*, and the value of *ρ_s_* is as follows [61]:

(5) $$\rho_{S}=\frac{E_{C}}{J_{C}}\times {\left(\frac{J_{\varphi }}{J_{C}}\right)}^{n-1}+\rho_{0}$$

where *E_c_* = 10^−4^ V/m is the critical electric field. *ρ*_0_ = 10^−14^ Ω·m [61] is set to avoid non-convergence during the calculation. *J_φ_* is the hoop current density. The power index *n* is 21 [61]. *J_c_* is the critical current density, defined as follows [52]:

(6) $$J_{C}\left(B,T\right)=\frac{J_{c0}}{{\left(1+\frac{\sqrt{k^{2}B_{z}^{2}+B_{r}^{2}}}{B_{0}}\right)}^{\beta_{0}}}{\left[1-{\left(\frac{T}{T_{c}}\right)}^{2}\right]}^{\frac{3}{2}}$$

where *B_r_* and *B_z_* are the radial and axial magnetic flux densities, respectively. The other parameters are set as follows: *J_c_*_0_ = 1.56 × 10^11^A/m^2^, *k* = 0.3, *B*_0_ = 0.02 T, and *β*_0_ = 0.6.

The electromagnetic model is implemented using the PDE module in COMSOL. The same applied current is assigned to each tape using the pointwise constraint. After the start of quench, the sum of the integral of the local *J_φ_* in each REBCO, copper, and Hastelloy layer equals the total current of one turn *I*(*t*). The governing equation is as follows [52]:

(7) $$\int_{S}J_{\varphi }dS=I(t)$$

We assume the air domain is infinite, so the magnetic field boundary condition of the air is written as *H_r_* = 0, *H_z_* = 0.

Because of the dissipated power density *E_φ_J_φ_*, the temperature distribution in the REBCO pancake coil is described by the heat diffusion equation. We apply adiabatic conditions on all boundaries of the coil; the heat diffusion equation is given by:

(8) $$\gamma c_{p}\frac{\partial T}{\partial t}=\frac{1}{r}\frac{\partial }{\partial r}\left(rk\frac{\partial T}{\partial t}\right)+\frac{\partial }{\partial z}\left(k\frac{\partial T}{\partial z}\right)+Q_{J}$$

where *γ* is the mass density. *c_p_* and *k* represent the specific heat capacity and the thermal conductivity, respectively, which are temperature-dependent, as shown in Figure 2. *Q_J_* is the Joule heat:

(9) $$Q_{J}=\rho J_{\varphi }^{2}$$

(10) $$J_{\varphi }=\frac{\partial H_{r}}{\partial z}-\frac{\partial H_{z}}{\partial r}$$

A uniform temperature distribution (*T*_0_ = 20 K) inside the coil is set as the initial condition.

It is noteworthy that during quench propagation, non-negligible stress and strain are generated because of the combined action of the Lorentz force and the temperature rise. A 2D axisymmetric elastic mechanical model is implemented in COMSOL to handle the stress–strain calculation of the coil during quench propagation. To simplify the computations, several approximations are applied in this study. (1) The magnetic field change and heat generation caused by structural deformation are not taken into account, and geometric nonlinearity is neglected. (2) The constituent materials are considered to be elastically isotropic and continuous, with the parameters shown in Table 1. (3) It is assumed that all layers are bonded together and that there is perfect bonding between any two in-contact surfaces. (4) Axial displacement constraint conditions are imposed on the upper and bottom edges of the pancake coil, i.e., *v*(*z* = 0, *z* = h) = 0. (5) The radial stresses on the inner radius and outer radius boundaries are set to zero, i.e., *σ_r_*(*r* = R_IR_, *r* = R_OR_) = 0.

Based on the theory of elastic mechanics, the equilibrium and geometric equations of the 2D axisymmetric model can be expressed as:

(11) $$\left\{\begin{array}{c} \rho \frac{\partial^{2}u}{\partial t^{2}}=\frac{\partial \sigma_{r}}{\partial r}+\frac{\sigma_{r}-\sigma_{\varphi }}{r}+\frac{\partial \tau_{rz}}{\partial r}+f_{r}\\ \rho \frac{\partial^{2}v}{\partial t^{2}}=\frac{\partial \sigma_{z}}{\partial z}+\frac{\tau_{rz}}{r}+\frac{\partial \tau_{rz}}{\partial r}+f_{z} \end{array}\right.$$

(12) $$\varepsilon_{r}=\frac{\partial u}{\partial r},\varepsilon_{\varphi }=\frac{u}{r},\varepsilon_{z}=\frac{\partial v}{\partial z},\gamma_{zr}=\frac{\partial u}{\partial z}+\frac{\partial v}{\partial r}$$

where *u* and *v* are the radial and axial displacements, respectively. *ε_r_*, *ε_φ_*, *ε_z_,* and *γ_rz_* denote the strain components. *σ_r_*, *σ_φ_*, *σ_z_*, and *τ_rz_* are the stress components. *f_r_* and *f_z_* represent the radial and axial electromagnetic body forces, respectively.

(13) $$\left\{\begin{array}{c} f_{r}=JB_{z}=\mu_{0}H_{z}\left(\frac{\partial H_{r}}{\partial z}-\frac{\partial H_{z}}{\partial r}\right)\\ f_{z}=-JB_{r}={-\mu }_{0}H_{r}\left(\frac{\partial H_{r}}{\partial z}-\frac{\partial H_{z}}{\partial r}\right) \end{array}\right.$$

The constitutive relationships of the stress–strain for the constituent materials are as follows.

(14) $$\left[\begin{array}{c} \begin{array}{c} \varepsilon_{r}\\ \varepsilon_{\varphi } \end{array}\\ \begin{array}{c} \varepsilon_{z}\\ \gamma_{rz} \end{array} \end{array}\right]=\left[\begin{array}{cccc} \frac{1}{E} & -\frac{v}{E} & -\frac{v}{E} & 0\\ -\frac{v}{E} & \frac{1}{E} & -\frac{v}{E} & 0\\ -\frac{v}{E} & -\frac{v}{E} & \frac{1}{E} & 0\\ 0 & 0 & 0 & \frac{1}{G} \end{array}\right]\left[\begin{array}{c} \sigma_{r}\\ \sigma_{\varphi }\\ \sigma_{z}\\ \tau_{rz} \end{array}\right]+\left[\begin{array}{c} \alpha \\ \alpha \\ \alpha \\ 0 \end{array}\right]\left(T-T_{0}\right)$$

where *α* is the thermal expansion coefficient; *E* is the elastic modulus components; *υ* is Poisson’s ratio; *G* is the Lamé’s constants, *G* = *E*/(2(1 + *υ*)).

### 2.2. Electrical Circuit Model

After the quench of the coil, the normal zone gets overheated easily, and an internal overvoltage emerges during a general quench process without a suitable quench protection circuit. For a REBCO pancake coil, using a cold resistor in series with a cold diode is a primary method to avoid overheating, overstress, and internal overvoltage. The coil is operated at 150 A (0.8 *I_c_*_0_) initially. After the quench protection circuit is triggered, the coil forms a loop circuit with the dump resistor and diode, and the circuit equation is given by (15):

(15) $$L\frac{dI\left(t\right)}{dt}+\left[R_{d}+R\left(t\right)\right]I\left(t\right)=0$$

where *L* is the inductance of the coil; *I*(*t*) is the operating current after the quench protection circuit is triggered; *R_d_* is the parallel dump resistor; *R*(*t*) is the coil resistor after quenching, which is negligible as *R*(*t*) << *R_d_*.

### 2.3. Selection of the Dump Resistor

The selection criteria for the dump resistor should consider the following two factors: the dissipation velocity must be high, and the discharge voltage of the coil must be less than the safety value. An MIITs computation is used to select the value of the dump resistor [40,46]:

(16) $$F\left(T_{HS}\right)=\int_{0}^{T_{HS}}\left[\frac{C\left(T\right)}{\rho \left(T\right)}\right]dT=\int_{0}^{\infty }J{\left(t\right)}^{2}dt$$

where *F* is the MIITs; *C*(*T*) is the heat capacity; *ρ* is the material resistivity; *J*(*t*) = *I*(*t*)/*A* and *I*(*t*) = *I*_0_exp(-*R_d_*/*L × t*). *T_HS_* is the hot spot temperature. The function of the integral of *J*^2^dt is as follows:

(17) $$\int_{0}^{\infty }J{\left(t\right)}^{2}dt=\frac{L}{R_{d}}\frac{I_{0}^{2}}{2A^{2}}$$

where *L* is the inductance; *R_d_* is the parallel dump resistor; *I*_0_ is the operating current; *A* is the area of the coil. The above formulas show the relationship between *T_HS_* and *R_d_*. We choose a hot spot temperature of less than 200 K to avoid critical thermal stress [62]. According to Equations (16) and (17), we calculate the numerical value range of the dump resistor to be *R_d_* > 0.4 mΩ. We ultimately choose *R_d_* = 0.6 mΩ. The set value meets the withstand voltage limit, i.e., *R_d_* ≤ *U*_0_/*I*_0_, where *U_0_* represents the allowable voltage.

## 3. Results and Optimization

### 3.1. Effects of Trigger System

The sudden and excessive Joule heating causes serious damage to superconducting coils when a quench occurs. Therefore, quench protection is one of the most important issues for safe operation. The time taken to trigger the system dump *t_p_* (including switching actions) is essential. In this subsection, we will study the influence of *t_p_* on the temperature, current, and mechanical responses. We will then compare the mechanical responses with those obtained without implementing protection schemes, i.e., *t_p_* = ∞.

**Figure 3:**
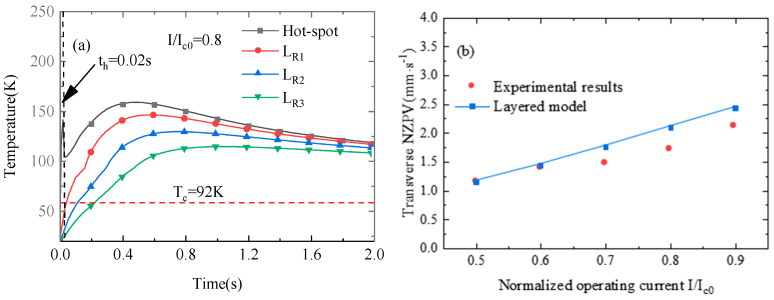
(**a**) Waveforms for the peak temperatures of the hot spot and probes *L_R_*_1_–*L_R_*_3_ and (**b**) the variation of NZPV with normalized operating current.

Figure 3 shows the variations in the temperatures of the hot spot and probes *L_R_*_1_–*L_R_*_3_ and the variation of NZPV with normalized operating current. From Figure 3a, it can be observed that the temperature of the hot spot will rapidly rise during the thermal triggering stage. The temperature of each point reaches its maximum value during the quench protection process and then decreases. According to the formula NZPV = ∆*w*/∆*t*, we take the time delay between *L_R_*_1_ and *L_R_*_3_ as ∆*t*, where ∆*w* represents the distance between *L_R_*_1_ and *L_R3_*. It can be seen that the simulation result for NZPV of the coil is close to the experimental results in Ref. [63]. The model in this paper is proved to be effective in predicting the quench characteristics of REBCO insulation coil.

**Figure 4:**
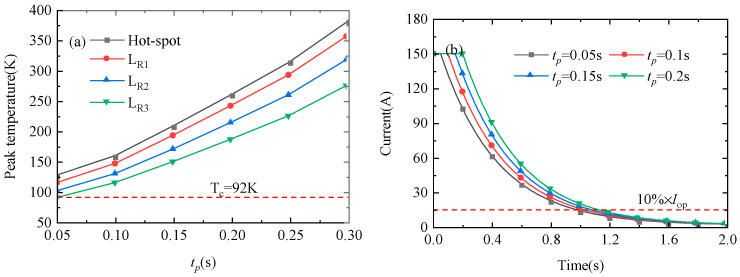
(**a**) Waveforms for the peak temperatures of the hot spot and probes *L_R_*_1_–*L_R_*_3_ and (**b**) the current after the quench protective action is triggered as *t_p_* varies.

Figure 4a shows the variations in the peak temperatures of the hot spot and probes *L_R_*_1_–*L_R_*_3_ and the variation of NZPV with normalized operating current. As seen in Figure 4a, the peak temperatures of the hot spot and probes *L_R_*_1_–*L_R_*_3_ increase significantly as *t_p_* increases for a given value of the input energy, and a coil is safer from quench damage for a smaller value of *t_p_*. Figure 4b shows the waveform of the coil current after the quench protective action is triggered as *t_p_* varies. To simulate the powering-off of the DC current source during quench protection, the operating current is decreased to 0 A at *t* = 0.05 s, *t* = 0.1 s, *t* = 0.15 s, and *t* = 0.2 s. The decrease in the operating current to 10% × *I_0_* is taken as the reference value for the end of the release time. We find that the increase in *t_p_* has no influence on the dissipation velocity. From Equation (15), it can be seen that the current during the quench protection process is related to the coil self-inductance, the parallel dump resistor, and the coil resistor after quench. When the coil resistor after quench is ignored, the remaining amount is constant, so *t_p_* will not affect the dissipation velocity.

**Figure 5:**
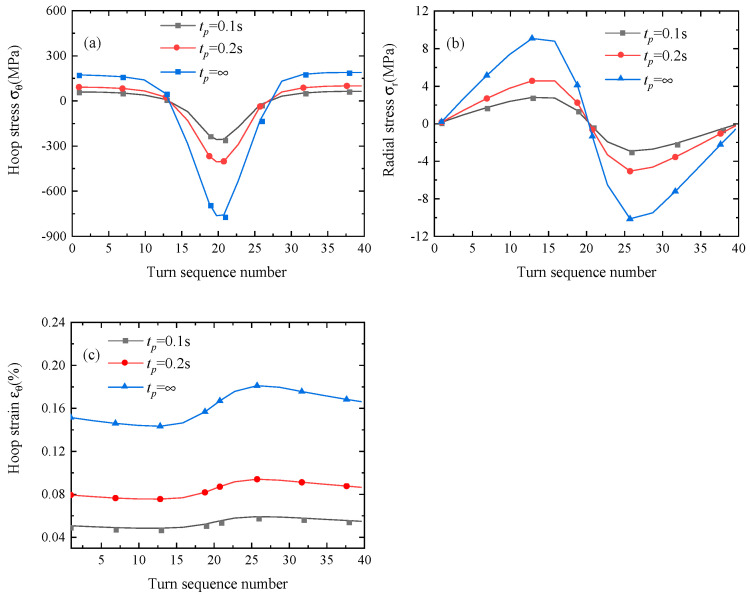
Distributions of (**a**) the hoop stress, (**b**) the radial stress, and (**c**) the hoop strain along the radial direction of the coil during the quenching process when *t_p_* is 0.1 s, 0.2 s, and ∞.

In order to clarify the effects of *t_p_* on the stress and strain distribution, the profiles of the hoop and radial stresses and hoop strain in the middle point of the copper layers are plotted in Figure 5, with the stress and strain distributions approximately uniform along the tape width.

It is found from Figure 5 that the hoop and radial stresses and hoop strain increase greatly with increasing time taken triggering the system dump. As *t_p_* goes from 0.1 s to 0.2 s and then to ∞, the peak compressive hoop stress rises from −266.76 MPa to −415.52 MPa and then to −777.36 MPa, and the corresponding peak tensile radial stress climbs from 2.68 MPa to 4.47 MPa and then to 9.05 MPa. The peak tensile radial stress without quench protection is close to the critical transverse tensile stress of ~10 MPa in Ref. [64], which is likely to cause delamination. The peak tensile hoop strain rises from 0.057% to 0.18% with *t_p_* increasing from 0.1 s to ∞. Thus, we can see that the peak stress and strain can be decreased greatly by implementing protection schemes in time. As a consequence of the low transverse quench propagation of the HTS coil, localized high-temperature gradients and temperature rises may occur. Consequently, the hoop and radial stresses and hoop strain vary greatly with an increasing radius near the heat region. Except for several copper layers near the heat region, the remaining ones are subjected to tensile hoop stress. The maximum tensile hoop stress, radial stress, and radial strain are found on the innermost copper layer, the copper layer on the left side of the heat region, and the copper layer on the right side of the heat region, respectively.

### 3.2. Effects of Background Field

In this subsection, the stress–strain states of the HTS coil under high field conditions during the quench process are evaluated and compared.

**Figure 6:**
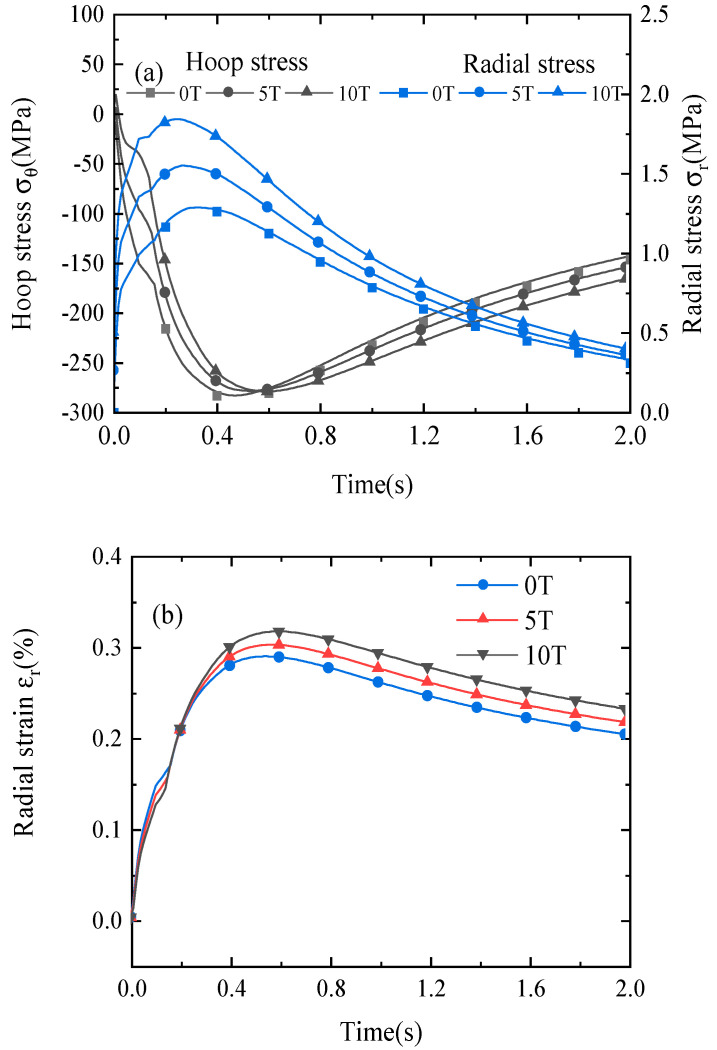
The variation of (**a**) the hoop and radial stresses and (**b**) the radial strain in the REBCO layer (probe *L_R_*_3_) with axial background fields of 0 T, 5 T, and 10 T during the quench process.

Figure 6 shows the variation in the hoop stress, radial stress, and radial strain of probe *L_R_*_4_ with axial background fields of 0 T, 5 T, and 10 T. The hoop and radial stresses drastically increase after the quench due to the large Joule heat. After the quench protection process, they reach their maximum values and then gradually decrease, as shown in Figure 6a. The radial stresses are in the tensile state after the heat pulse, and the hoop stresses are almost all in the compressive state. For the case without an external field, the hoop and radial stresses are mainly determined by the thermal stress due to the localized high-temperature gradient and temperature rise near the heat region and reach maximum values of −287.03 Mpa and 1.27 Mpa, respectively. For the case with axial background fields of 5 T and 10 T, the peak radial stress rises from 1.27 Mpa to 1.54 Mpa and then to 1.84 Mpa because of the large radial Lorentz force of *f_r_* = *JB_z_*. The axial background field has no great influence on the peak hoop stress after protection schemes are implemented. From Figure 6b, one can see that the radial strains reach their maximum values during the quench process, and the peak radial strain increases from 0.29% to 0.3% and then to 0.32% with axial background fields of 5 T and 10 T, respectively.

In summary, the stress and strain distribution is affected by both the temperature and magnetic field. The radial and hoop stresses and radial strain reach their maximum values during the quench process, and the axial background field has a significant influence on the radial stress.

The strain-based quench detection method has proven to be effective in low-temperature superconducting magnets [65,66]. The temperatures and radial strains in the superconducting layers of a REBCO pancake coil rise with a similar trend after the heat pulse [38], which inspires us to determine whether the radial strain rates can characterize quench propagation in a REBCO coil.

**Figure 7:**
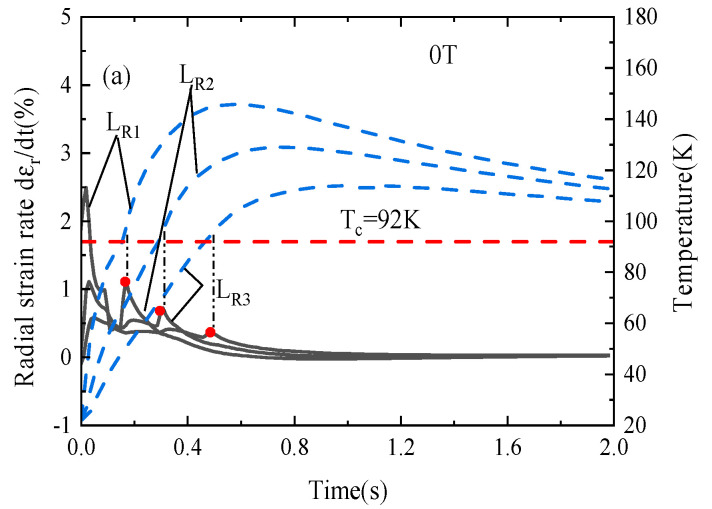

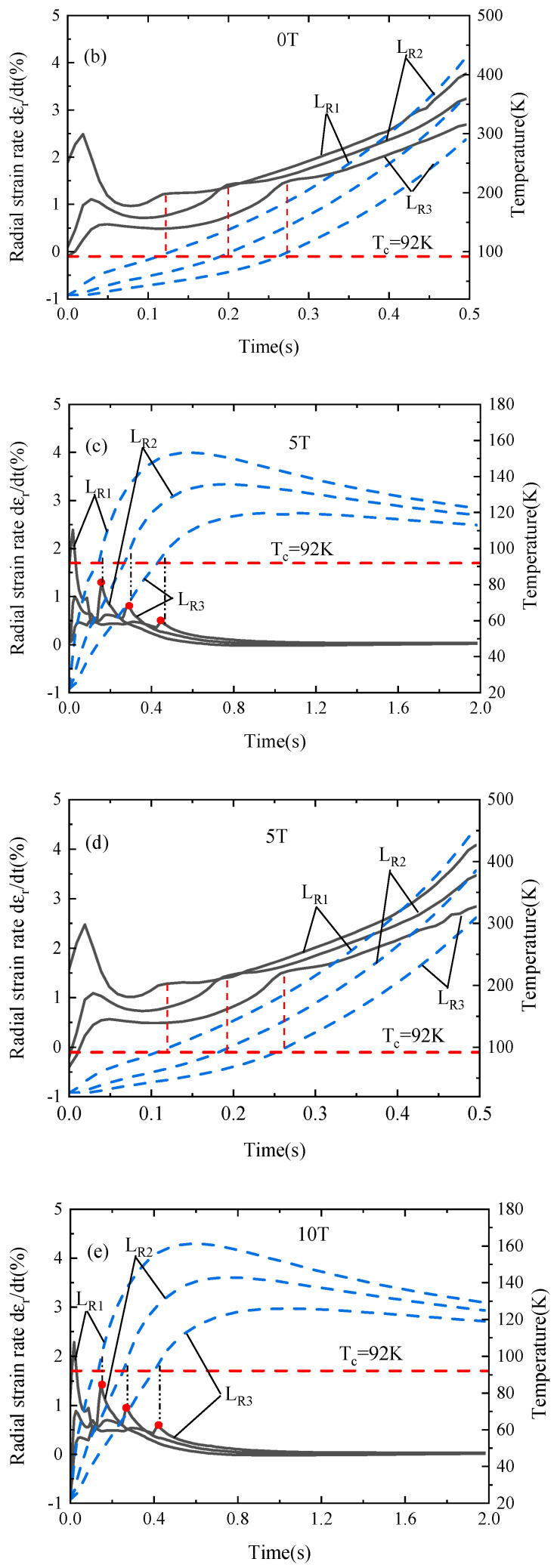

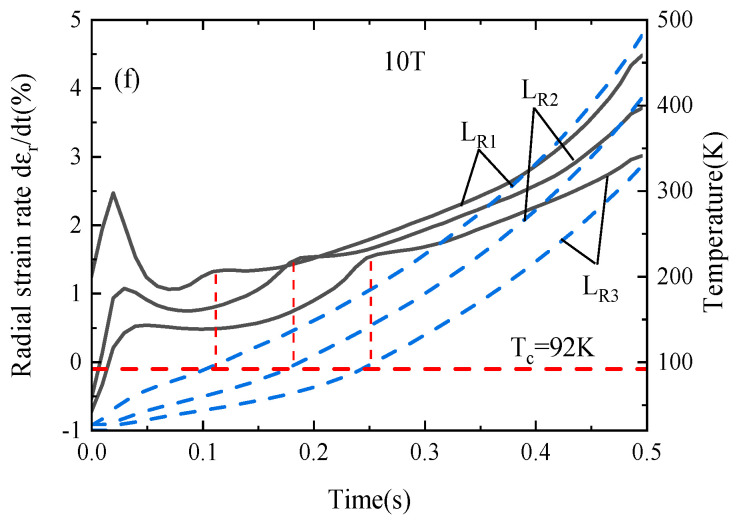
The variation in the radial strain rate and temperature of probes *L_R_*_1_, *L_R_*_2_, and *L_R_*_3_ with axial background filed of 0 T (**a**), 5 T (**c**), and 10 T (**e**) during quench protection, and 0 T (**b**), 5 T (**d**), and 10 T (**f**) during quench propagation.

Figure 7 compares the variations of the temperature and radial strain rate of probes *L_R1_*–*L_R_*_3_ with axial background fields of 0 T, 5 T, and 10 T during quench protection. Two peaks in the radial strain rate occur during the quench process, as shown in Figure 7a,c,e. The former peak is contributed to the heat pulse, and the latter peak almost coincides with the maximum temperature rise rate, which is near the critical temperature occurrence time. When the temperature continues to decrease, the strain rate becomes negative. To more clearly demonstrate the relationship between the radial strain rate and the quench, in Figure 7b,d,f, we show the radial strain rate at 0.5 s without protection schemes implemented. After the first peak generated by the heat pulse, we can find that, with the time, the strain rate has an apparent slope change. For comparison, the temperature changes at these locations are also plotted in the figure. It is clearly found that the instant time of an apparent slope change of the radial strain rate is just right and consistent with the onset time of a quench of the REBCO coil determined by the critical temperature (i.e., Tc = 92 K), which implies that the change in strain rate could be a way of detecting the quench occurrence. Instead of recording temperature during a quench process, the strain rate measurement can be utilized as a way to detect a quench that has been observed. The experimental measurements on superconducting solenoid magnets at cryogenic temperature in the previous work supported such strain rate behaviors [66]. In Ref. [38], numerical simulation work also verified the theoretical feasibility of strain-based high-temperature superconducting coil quenching detection technology. Meanwhile, it can be observed that the relationship between strain rate and quench is not affected by the background field. At present, the detection method for the high-temperature superconducting magnet is relatively simple, such as temperature, and the strain rate detection method provides a possibility.

These numerical results show that quench detection by observing the slope change of the radial strain rate is feasible regardless of the background field, which also ensures the theoretical feasibility of the strain-based quench detection method for a REBCO pancake coil under different fields.

### 3.3. Optimization

The material properties of the insulation layer can significantly affect the quench behavior of HTS magnets. Here, we examine the influences of the thermal conductivity and thermal expansion coefficient of the insulation layer on the hoop stress and hoop strain in the REBCO layer, Hastelloy substrate layer, copper stabilizer layer, and insulation layer during quench protection through parametric analysis.

**Figure 8:**
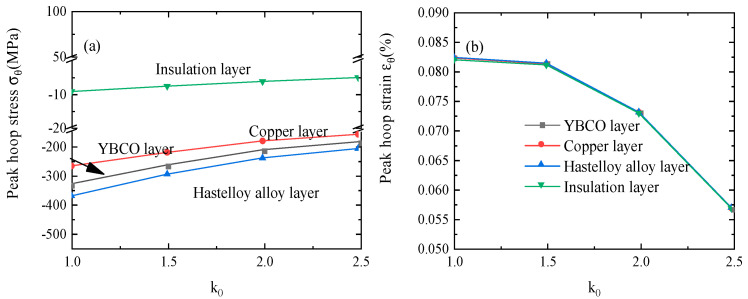
The variation in (**a**) the peak hoop stress and (**b**) the peak hoop strain with increasing thermal conductivity during the quenching process.

We study the effect of the thermal conductivity of the insulation layer on the stress–strain state and perform a parametric analysis by introducing scale coefficient *k*_0_:

(18) $$k_{0}=\frac{k\left(T\right)}{k_{kapton}\left(T\right)}$$

where *k_kapton_* is shown in Figure 2a. From Figure 8, one can see that the peak hoop stress and strain of each layer decrease greatly as the thermal conductivity increases. It is easy to understand that the quench behavior of HTS magnets is significantly improved if the turn-to-turn electrical insulation is thermally conducting. The turn-to-turn quench propagation is increased by increasing the thermal conductivity.

**Figure 9:**
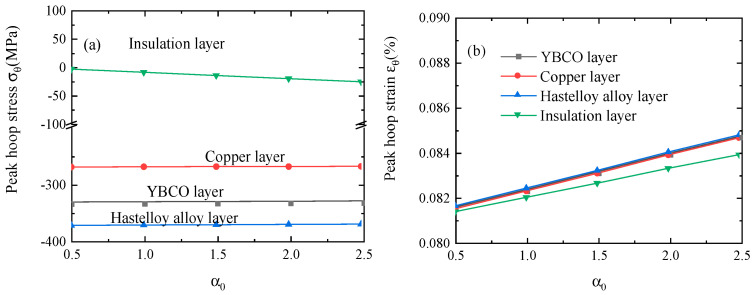
The variation in (**a**) the peak hoop stress and (**b**) the peak hoop strain with an increasing thermal expansion coefficient during the quench process.

Furthermore, we introduce the scale coefficient *α*_0_ to study the effect of the thermal expansion coefficient of the insulation layer on the peak stress and strain of each layer.

(19) $$\alpha_{0}=\frac{\alpha \left(T\right)}{\alpha_{kapton}\left(T\right)}$$

where *α_kapton_* is shown in Table 1. The peak hoop stress of the insulation layer increases significantly as the thermal expansion coefficient increases, as shown in Figure 9. The peak hoop strain of each layer increases to a certain extent with the increase in the thermal expansion coefficient. It can be concluded that decreasing the thermal expansion coefficient of the insulation layer can suppress the maximum stress and strain, though the effect is limited.

The delamination between constituent layers of the REBCO pancake coil occurs after the transverse tension exceeds the critical tension. The variations in the thickness of the copper layer and inner coil radius may significantly affect the peak stresses and strains in REBCO pancake coils. In this section, the effects of these two design factors are investigated.

**Figure 10:**
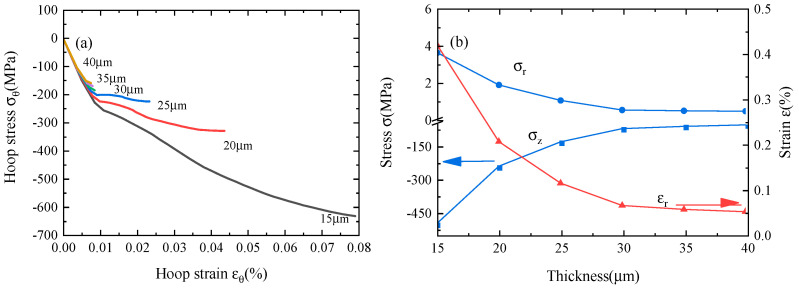
(**a**) The hoop stress–strain curves before the stresses reach the maximum value, and (**b**) the radial stress, axial stress, and radial strain for different copper thicknesses during the quench process.

Hoop stress–strain curves are obtained for coils with different copper contents, as shown in Figure 10a. One can see that an increased copper thickness results in a softening of the coil. Figure 10b shows the variations in the peak radial stress, peak axial stress, and peak radial strain with the thickness of the copper layer. The peak stresses and strain decrease significantly as the copper thickness increases from 15 μm to 25 μm. Continuing to increase the thickness of the copper layer can suppress the maximum stresses and strain, although this effect is limited. This is easy to understand because increasing the copper thickness can enhance the NZPV. According to the results in Figure 10, the greatest copper layer thickness practically available on the conductor of choice is of the order of 25 μm to 30 μm.

**Figure 11:**
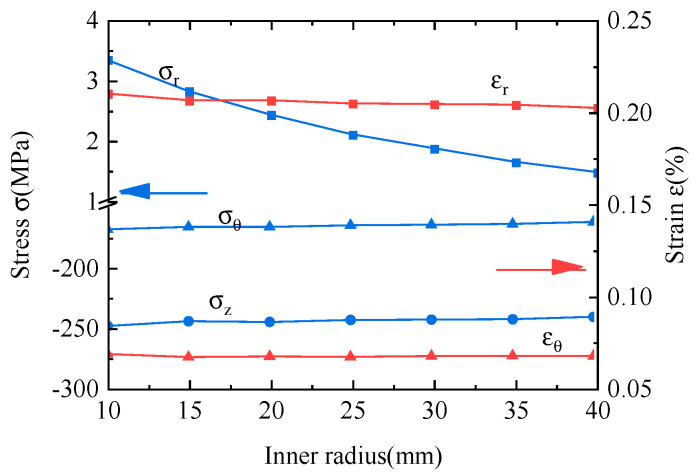
The variation of the radial stress, hoop stress, axial stress, radial strain, and hoop strain with the inner coil radius during the quenching process.

We next study the effects of the inner coil radius on the peak stresses and strains, as shown in Figure 11. The peak radial stress decreases significantly as the inner coil radius increases. The peak radial stress decreases from 3.34 MPa to 1.47 MPa as the inner coil radius changes from 10 mm to 40 mm. Increasing the inner coil radius can also suppress the peak axial and hoop stresses and radial and hoop strains, though this effect is very limited. Increasing the inner coil radius can increase the NZPV and reduce the localized high-temperature gradient and temperature rise. In summary, increasing the inner coil radius reduces the radial stress.

## 4. Conclusions

In this paper, a 2D axisymmetric electro–magneto–thermo–mechanical coupling model has been developed to study the mechanical responses and electro–thermal characteristics of a REBCO pancake coil during quench protection. The following conclusions can be drawn.

(1) The decrease in the time taken to trigger the system dump can significantly decrease the temperature but has no influence on the dissipation velocity. After the quench protection process, the maximum tensile radial stresses are less than the critical transverse tensile stress.
(2) The radial and hoop stresses and radial strain reach their maximum values during the quench process, and the axial background field has a great influence on the radial stress.
(3) An apparent slope change of the radial strain rate is observed when the quench occurs regardless of the background field, which suggests the theoretical feasibility of a strain-based quench detection method for REBCO pancake coils.
(4) The peak hoop stress and strain of each layer decrease greatly as the thermal conductivity of the insulation layer increases, and decreasing the thermal expansion coefficient of the insulation layer can suppress the maximum stress and strain, though this effect is limited. Increasing the thickness of the copper layer can suppress the maximum stresses and strain to a degree. Increasing the inner coil radius can also reduce the radial stress.

## Figures and Tables

**Table 1:** Properties of the constituent materials of the coil [52,59].

Property	Copper	REBCO	Hastelloy	Kapton
Mass density *γ* (kg/m^3^)	8940	5900	8890	1420
Elastic modulus *E* (GPa)	Figure 2	157	Figure 2	30
Poisson ratio *υ*	0.343	0.3	0.307	0.3
Thermal expansion coefficient *α* × 10^−5^ (K^−1^)	1.67	1.34	1.09	2

## Data Availability

The original data supporting the research are not publicly available, but a portion of the data that is not confidential is available on request from the corresponding author.
